# Modelling effectiveness of herd level vaccination against Q fever in dairy cattle

**DOI:** 10.1186/1297-9716-42-68

**Published:** 2011-05-23

**Authors:** Aurélie Courcoul, Lenny Hogerwerf, Don Klinkenberg, Mirjam Nielen, Elisabeta Vergu, François Beaudeau

**Affiliations:** 1 INRA, UMR1300 Bio-agression, Epidémiologie et Analyse de Risque, Atlanpole La Chantrerie, BP 40706, 44307 Nantes, France; 2 LUNAM Université, Oniris, UMR1300 Bio-agression, Epidémiologie et Analyse de Risque, Atlanpole La Chantrerie, BP 40706, 44307 Nantes, France; 3 Faculty of Veterinary Medicine, Utrecht University, Yalelaan 7, 3584 CL Utrecht, The Netherlands; 4 INRA, UR341 Mathématiques et Informatique Appliquées, Domaine de Vilvert, 78350 Jouy-en-Josas, France

## Abstract

Q fever is a worldwide zoonosis caused by the bacterium *Coxiella burnetii*. The control of this infection in cattle is crucial: infected ruminants can indeed encounter reproductive disorders and represent the most important source of human infection. In the field, vaccination is currently advised in infected herds but the comparative effectiveness of different vaccination protocols has never been explored: the duration of the vaccination programme and the category of animals to be vaccinated have to be determined. Our objective was to compare, by simulation, the effectiveness over 10 years of three different vaccination strategies in a recently infected dairy cattle herd.

A stochastic individual-based epidemic model coupled with a model of herd demography was developed to simulate three temporal outputs (shedder prevalence, environmental bacterial load and number of abortions) and to calculate the extinction rate of the infection. For all strategies, the temporal outputs were predicted to strongly decrease with time at least in the first years of vaccination. However, vaccinating only three years was predicted inadequate to stabilize these dynamic outputs at a low level. Vaccination of both cows and heifers was predicted as being slightly more effective than vaccinating heifers only. Although the simulated extinction rate of the infection was high for both scenarios, the outputs decreased slower when only heifers were vaccinated.

Our findings shed new light on vaccination effectiveness related to Q fever. Moreover, the model can be further modified for simulating and assessing various Q fever control strategies such as environmental and hygienic measures.

## Introduction

Q fever is a zoonotic disease caused by *Coxiella burnetii*, a bacterium found worldwide in a wide range of animals. In ruminants, the infection may cause abortions, infertility, metritis or chronic mastitis [1-4], which can lead to non negligible economic losses for the infected herds. Furthermore, since 2007, Q fever has become an important public health problem in several parts of Europe [5-7]. Although Q fever is asymptomatic in 60% of human cases, it can lead to acute or chronic infections and cause flu-like syndrome, hepatitis, pneumonia, endocarditis or abortions [8]. In the Netherlands, where a steep increase in the number of human cases was observed in 2007, 2008, and 2009, a link has been established between some human cases and farms of small ruminants where abortions due to Q fever were detected [9]. Ruminants are indeed recognized as the main source of human infection [10,11]. Infected animals shed large quantities of bacteria into the environment through faeces, vaginal mucus, urine, milk and especially parturition products [4,12,13]. *C. burnetii *survives very well in the environment and contaminates aerosols and dust [14]. These infected particles are the main route of infection for both animals and humans. Due to its importance in both animal and public health, the control of this infection is crucial. Therefore, any control measure leading to a decrease in the prevalence of shedders and in the environmental bacterial load seems a key point to limit both the spread of the infection in ruminants and the zoonotic risk.

Nowadays, in infected cattle herds in France, control measures against Q fever consist of environmental measures such as destruction of placentas or disinfection of birth locations, antibiotic treatment like oxytetracycline injections during the last month of gestation, and vaccination [15]. According to Rodolakis et al. [15], vaccination would be an efficient tool to control the disease. A phase I vaccine was indeed shown to prevent abortions and dramatically reduce the frequency of bacterial shedding in the milk, vaginal mucus and faeces [16]. Besides, according to Guatteo et al. [17], susceptible cattle that were vaccinated when non pregnant had a five times lower probability to become a shedder than an animal receiving placebo.

Thus, in the field, vaccination is often recommended in infected herds after the occurrence of abortions due to Q fever. However, the studies assessing the vaccination efficacy in ruminants were carried out in experimental conditions or for a limited period of time and they evaluated the effect of the vaccine mostly at the individual level. Therefore, it is difficult to extrapolate these results to the case of a whole herd vaccination over several years. Furthermore, different vaccination strategies can be implemented: the duration of the vaccination programme as well as the category of vaccinated animals (e.g., the whole herd or the heifers only) have to be determined. To assess the long run effectiveness of these different strategies in reducing the infection prevalence or the environmental bacterial load, field studies are not optimal: no reference situation (without control strategy) is generally available, and long-term observations must be performed, making these studies very costly and even unfeasible. Modelling is therefore a convenient approach as it provides means to compare the effectiveness of different potential management strategies [18].

The objective of this model study was to assess the comparative effectiveness of several vaccination strategies against *C. burnetii *in an already infected dairy cattle herd. The criteria considered for efficacy evaluation were changes in the prevalence of shedders, the environmental bacterial load, the number of abortions, as well as the extinction rate of infection.

## Materials and methods

A model representing the *C. burnetii *infection dynamics in a standard French dairy cattle herd and different vaccination strategies was elaborated based on a previous variant model not including interventions. First of all, the epidemic model representing the natural course of infection (i.e. without any control strategy) will be briefly described, then the inclusion of vaccination will be presented and finally, the different vaccination scenarios that we tested will be explained in detail. Some elements of a sensitivity analysis performed to identify the most influent parameters and structural characteristics of both the model variants without and with vaccination are given in Additional file 1.

### General description of the epidemic model of the natural course of infection

**Figure 1:**
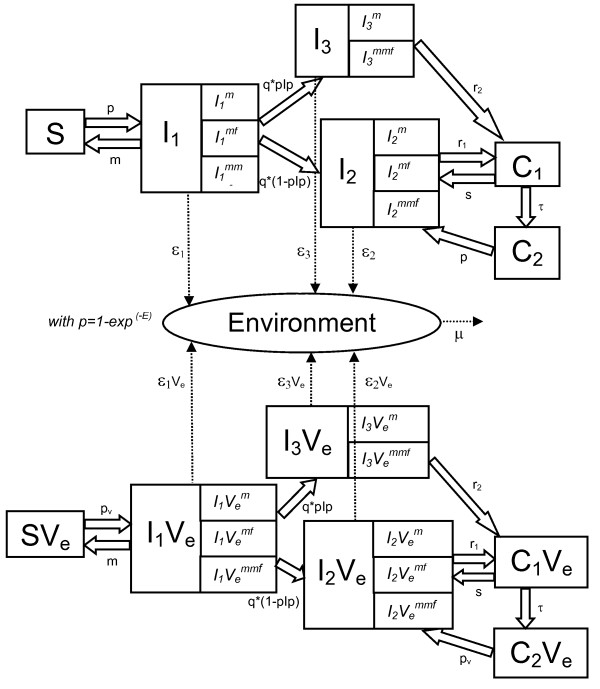
**Flow diagram describing the modelled spread of *C. burnetii *within a cattle herd**. The health states are the following: *S*, non-shedder apparently susceptible cow, *I*_*1*_, shedder which still has the possibility to eliminate the bacterium and to become *S *again, *I*_*2*_, shedder which no longer has the possibility to become *S *again, *I*_*3*_, shedder which no longer has the possibility to become *S *again and sheds in milk in a persistent way, *C*_*1*_, non-shedder but still infected individual and *C*_*2*_, non-shedder which was *C*_*1 *_in the past but eliminated the bacterium. The *V*_*e *_states (*SV*_*e*_*, I*_*1*_*V*_*e*_*, I*_*2*_*V*_*e*_*, I*_*3*_*V*_*e*_*, C*_*1*_*V*_*e *_and *C*_*2*_*V*_*e*_) are defined in the same way as *S*, *I*_*1*_, *I*_*2*_, *I*_*3*_, *C*_*1 *_and *C*_*2 *_respectively, except that these animals have been vaccinated when susceptible and non pregnant and are then assumed "vaccinated in an effective way" (*V*_*e*_*)*. *I *and *IV*_*e *_cows are in the subcategory *m *if they shed in milk only, *mf *if they shed in vaginal mucus/faeces only and *mmf *if they shed in milk and vaginal mucus/faeces. *E *represents the environmental bacterial load and *p*, the probability of infection or reinfection for non *V*_*e *_individuals, is equal to . *p*_*v *_is the probability of infection or reinfection for *V*_*e *_individuals, which is a fraction of *p*. The other model parameters are presented in Additional file 1: Table S1. *ε*_*1*_, *ε*_*2*_, *ε*_*3*_, *ε*_*1*_*V*_*e*_, *ε*_*2*_*V*_*e *_*and ε*_*3*_*V*_*e *_are the quantities of bacteria shed during a time step by an individual *I*_*1*_, *I*_*2*_, *I*_*3*_, *I*_*1*_*V*_*e*_, *I*_*2*_*V*_*e *_and *I*_*3*_*V*_*e *_respectively and contaminating the environment. For a any shedder, *ε *represents the sum, for each shedding route, of the quantity of bacteria released, *Qty*, times *ρ*, its fraction reaching the herd environment.

The model represents the spread of the bacterium in a dairy herd of lactating and dry cows (diagram flow in Figure 1 and parameters in Additional file 1: Table S1). It is a stochastic individual-based model in discrete time with a time step of one week. The model also represents the heterogeneity of shedding described for *C. burnetii *infections [13,19,20]: in field data [20], differences in shedding routes, levels (i.e. quantities of bacteria shed) and duration were observed between animals. Some animals can also become chronically infected [12]. Therefore, in the model, three types of shedders with different shedding characteristics and epidemiological behaviours were distinguished: (i) shedders of type 1 which have the possibility to clear the infection (*I*_*1*_), (ii) shedders of type 2 which are chronically infected (*I*_*2*_), and (iii) shedders of type 3 which are chronically infected and always shed in milk, for a longer period of time and at higher levels than shedders of type 2 (*I*_*3*_). By inhaling bacteria contained in the environment, a susceptible cow (*S*) can become infectious and start shedding. Either it manages to eliminate the bacterium and becomes apparently susceptible again (i.e. non-shedder without antibodies) or it becomes a chronically infected shedder (*I*_*2 *_or *I*_*3*_). Since the shedding is intermittent [12], these types of shedders can stop shedding and start again. We assume that a former *I*_*2 *_or *I*_*3 *_also has a chance to clear the infection. However, if this cow is infected again, it becomes a chronically infected animal again. Therefore, each cow is in one of the six mutually exclusive health states at a given time: *S *(non-shedder apparently susceptible cow), *I*_*1 *_(shedder of type 1), *I*_*2 *_(shedder of type 2), *I*_*3 *_(shedder of type 3), *C*_*1 *_(non-shedder but still infected cow), *C*_*2 *_(non-shedder which was *C*_*1 *_in the past but cleared the infection). Besides, sub-categories are defined for the shedder cows with respect to the shedding route. Thus, an *I*_*1 *_or *I*_*2 *_cow can shed in (1) milk only (denoted by  or respectively), (2) vaginal mucus and/or faeces ( or respectively), or (3) milk and either vaginal mucus or faeces or both ( or respectively). In the same way, an sheds in milk only and an sheds in milk and vaginal mucus and/or faeces (by definition, an *I*_*3 *_animal always sheds in milk and can not be in the  state). The possible transitions between health states are represented in the top part of Figure 1. Shedders (*I*_*1*_, *I*_*2 *_and *I*_*3*_) fill the environment compartment (*E*) with bacteria. Three categories of shedding levels are represented: low, moderate and high level, corresponding respectively to a quantity of bacteria shed *Qty *of 1/3000, 1/30 and 1 unit of environment per week. The probability for a cow to shed at one of these levels depends on her infectious state (*I*_*1*_, *I*_*2 *_or *I*_*3*_) and on the shedding route (milk or mucus/faeces). This probability is governed by the probability distributions *Q*, described in Additional file 1: Table S1. Only a fraction *ρ *of the quantity of bacteria shed by a shedder is assumed to reach the environment. Thus, the quantity of bacteria arriving into the environment during a time step ***t ***is the sum, for all the shedding routes of all the shedders, of , the quantity of bacteria shed by the shedder ***i ***through the shedding route (*route *∈ {*milk*, *mucus*/*faeces*}), times *ρ*^*route *^the impact of this shedding on the environment (see Additional file 1: section 1.2. for details). The probability of infection or re-infection, *p *(transition from *S *to *I*_*1 *_or from *C*_*2 *_to *I*_*2*_) is expressed at each time step as  where *E*_*t *_is the quantity of bacteria in the herd environment at time *t *(one unit of *E*_*t *_corresponding to a probability of transition from *S *to *I*_*1 *_of (1 - 1/e)). The mortality rate of *C. burnetii *in the environment, *μ*, includes the natural mortality of the bacterium and its removal in relation to the periodic cleaning of the cattle housing carried out by the farmer.

Since abortions are the main clinical signs attributable to *C. burnetii *infections [14,20], they are also represented in the model: a cow is assumed to have a risk to abort after her first infection, her reinfection or the resumption of shedding (i.e. after a transition from *S *to *I*_*1*_, from *C*_*1 *_to *I*_*2 *_or from *C*_*2 *_to *I*_*2*_). According to Arricau-Bouvery et al. [21], aborting females in cattle generally do not abort during the following gestations. Based on [21], it was assumed in our model that a cow can abort only once in her life. If the cow aborts in the first or second third of gestation, she sheds a moderate quantity of bacteria *Qty *through the mucus/faeces, whereas if the abortion occurs in the last third of gestation, a high quantity of bacteria is released through this shedding route.

The epidemic model was also coupled to a model of population dynamics in order to represent the gestation and lactation cycles of each cow. In short, for each cow the lactation number is represented, as well as the stage of lactation, the stage of gestation, the abortion history, the health state and the shedding characteristics (if the cow is shedding).

### Representation of the vaccination

Based on Guatteo et al. [16], possibly due to downregulated Th1-type immune responses during pregnancy, we assumed that the vaccine is effective when applied to non pregnant uninfected individuals. Thus, in the epidemic model, non pregnant *S *and *C*_*2 *_individuals become protected when vaccinated and move into the "vaccinated in an effective way" (*V*_*e*_) state (bottom of Figure 1). Pregnant *S *and *C*_*2*_, as well as all *I*_*1*_, *I*_*2 *_and *C*_*1 *_are what we defined the uselessly vaccinated: the vaccine has no effect on the infection dynamics in these animals, and they keep moving between the states *S*, *I*_*1*_, *I*_*2*_, *I*_*3*_, *C*_*1 *_and *C*_*2 *_(top of Figure 1). Six additional health states are defined for the *V*_*e *_individuals. *SV*_*e *_and *C*_*2*_*V*_*e *_individuals can get infected and become *I*_*1*_*V*_*e *_or *I*_*2*_*V*_*e *_respectively with a decreased transition rate *p*_*v *_(equal to a fraction of *p*). Except for this difference between *p *and *p*_*v*_, the *V*_*e *_animals can evolve through the same health states with identical transition rates as the non *V*_*e *_animals.

Regarding the shedding levels, according to Guatteo et al. [16], the only quantified bacterium load of a *V*_*e *_shedder was lower than the lowest bacterium load of the placebo cows. Besides, in Rousset et al. [22], the bacterial loads in vaginal swabs were lower in vaccinated than in non vaccinated animals. Therefore, we assumed that no high level shedding is possible for *V*_*e *_animals and that the probability to shed at a low level is increased (expressed through probability distributions *QV*_*e *_in Additional file 1: Table S1). Finally, based on Arricau-Bouvery et al. [16], it was assumed that the *V*_*e *_cows cannot abort.

### Vaccination scenarios

#### Scenario 1: vaccination over the whole simulation period (10 years)

At the start of the simulation, all the cows are vaccinated and all the heifers entering the herd of cows are assumed to be *SV*_*e *_(susceptible and vaccinated when non pregnant). In addition, all the animals are boosted every year: there is no loss of immunity and no possible transition from the *V*_*e *_states to the non *V*_*e *_states.

#### Scenario 2: vaccination for a limited period of time (3 years)

The assumptions are the same as those of scenario 1 except for the vaccination duration. Here, the herd is supposed to be vaccinated for three years. At the end of this 3 year period, two assumptions regarding the evolution of immunity were explored.

▪ Scenario 2A: immunity lasts for one year. One year after the end of the vaccination period, the *V*_*e *_animals lose their immunity and move to the non *V*_*e *_equivalent states (e.g. an *I*_*2*_*V*_*e *_cow becomes an *I*_*2 *_cow).

▪ Scenario 2B: lifelong immunity. After the vaccination period, the *V*_*e *_animals do not lose their immunity and keep moving within the *V*_*e *_states until the end of their life.

#### Scenario 3: vaccination of the heifers only over the whole simulation period (10 years)

At the start of the simulation the cows are not vaccinated. They stay in the non *V*_*e *_states and progress through infection states. Only the heifers arriving thereafter are assumed to be vaccinated in an effective way. These animals are in the *SV*_*e *_state when entering the dairy herd. Afterwards, all the previously vaccinated animals are boosted every year: there is no loss of immunity and no transition from the *V*_*e *_states to the corresponding non *V*_*e *_states.

#### Negative control

No control programme was implemented and all the animals progress through the non *V*_*e *_states.

### Parameters and initial conditions

The values of all epidemiologic parameters are displayed in Additional file 1: Table S1. Parameters *m*, *q*, *r*_*1*_, *s *and *μ *were fixed at their values estimated using data from five French chronically infected dairy cattle herds [23]; probability distributions of shedding related parameters, *α*, *β*, *β*_*calv*_, *γ, γ*_*calv*_, (governing the partition in subcategories according to the shedding route) and *Q1, Q2, Q3, Q4 *and *Q5 *(characterizing the shedding levels), were qualitatively calibrated to match field data, as well as *pIp *(the proportion of new chronically infected shedders becoming I_3_) and *probav *(the probability of abortion due to Q fever). The parameters governing the demography and herd management (Additional file 1: Table S2) were chosen to represent a standard French dairy cattle herd.

The transition rate *p*_*v *_was parameterized using the hazard ratio for a vaccinated initially susceptible animal to become a shedder in Guatteo et al. [17], which is equal to 0.21 with a 95% confidence interval of 0.05-0.90. Thus, we performed the simulations with *p*_*v *_= 0.21*p*. However, given the large confidence interval of [16], in scenario 1 two additional values were also tested (*p*_*v *_= 0.05*p *and *p*_*v *_= 0.90*p*) in order to determine the influence of this parameter value on the model output.

We simulated 100 repetitions of the introduction of a primiparous *I*_*2 *_cow that has just calved into a fully susceptible herd of 50 cows to generate infected herds. We let the model run until three abortions had occurred during a period of 12 months to initiate reactive vaccination. This limit was motivated by the fact that testing for a large panel of abortive pathogens (including *C. burnetii*) is usually performed in France from the 3^rd ^abortion within the calving period. Thus, we obtained 100 so called "initial herds", different from each other. Then, for each initial herd, the three vaccination scenarios and the negative control scenario were run once over a 10-year simulation period.

### Outputs of the model

The mean prevalence of shedders, the number of abortions per herd per year and the environmental bacterial load were the model's dynamic outputs of interest. In addition, for each scenario, the rate of extinction over the 10 year simulation period was calculated as the ratio between the number of extinct trajectories and the total number of repetitions. The infection was assumed to be extinct when there were no more *I*, *IV*_*e*_, *C*_*1 *_and *C*_*1*_*V*_*e *_cows in the herd at the end of the simulation time.

## Results

### Description of the herds at the start of the vaccination strategy

At the start of simulations, the mean prevalence of shedders (over 100 initial herds) was equal to 28.2% (min: 0.0%, max: 59.2%) and the mean prevalence of milk shedders amounted to 12.9% (min: 0.0%, max: 32.7%). In a herd, 92.1% of the cows on average had been shedders for at least one time step (min: 58.0%, max: 100%). The mean environmental bacterial load was 0.34 units (min: 0.01, max: 1.17) and the herds consisted of 49.7 cows on average (min: 41, max: 58). The herds were rather recently infected: the mean time from the beginning of the infection was 58 weeks (min: 8, max: 154).

### Influence of the vaccination scenarios on the temporal model outputs

**Figure 2:**
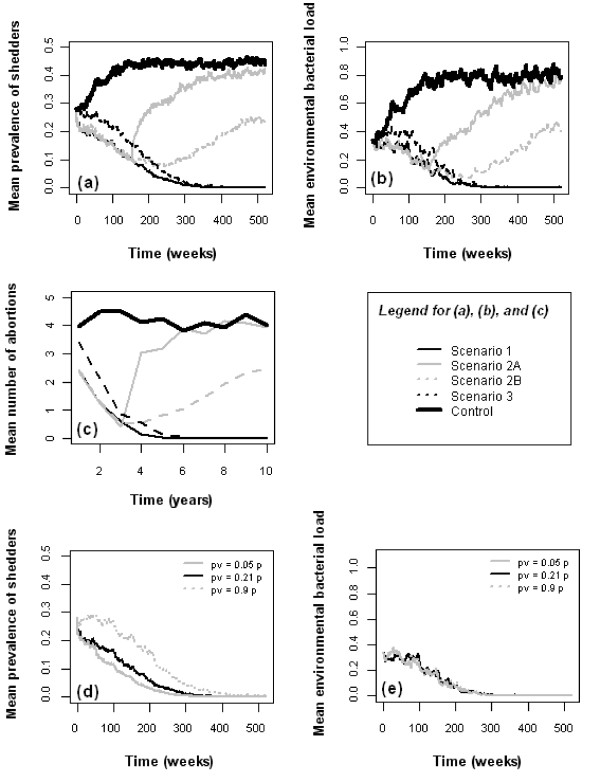
**Temporal dynamics of the mean prevalence of shedders**. (a), the mean environmental bacterial load (b) and the mean number of abortions (c) for the 4 vaccination scenarios. Scenario 1: vaccination of heifers and cows for a 10-year period (black line); scenario 2: vaccination of heifers and cows for a 3-year period with (scenario 2A - grey line) or without (scenario 2B - grey dotted line) loss of immunity one year after at the last vaccination; scenario 3: vaccination of heifers for a 10-year period (black dotted line); control: no vaccination (black thick line). Temporal dynamics of the mean prevalence of shedders (d) and mean environmental bacterial load (e) in scenario 1 with different values of *p*_*v *_(transition rate from *SV*_*e *_to *I*_*1*_*V*_*e*_).

If no control strategy was implemented, the mean prevalence of shedders, the mean environmental bacterial load and the mean number of abortions increased to a steady state around respectively 45% shedders, 0.8 unit of environment and four abortions per herd per year. On the contrary, for any vaccination scenario, all these outputs decreased with time at least for the first years of vaccination (Figure 2a, b, c). In scenario 1 (vaccination of heifers and cows during 10 years), the decrease covered the whole period. In scenario 3 (vaccination of heifers only for the whole simulation time), the decrease was much slower in the first years of vaccination than in scenario 1: the latter reached a mean prevalence of shedders of 5% and a mean environmental load of 0.05 respectively 9.2 and 9.9 months sooner than scenario 3. At the end of the vaccination period, the mean prevalence of shedders, environmental bacterial load and number of abortions were close to 0 in scenario 1 as well as in scenario 3. In scenario 2, there was an increase in the mean prevalence of shedders, the yearly number of abortions and the environmental bacterial load, after the vaccination ceased. For scenario 2A, this increase occurred immediately after the loss of immunity, whereas for scenario 2B (lifelong immunity), the increase was almost zero in the first year without vaccination and more progressive afterwards. Thus, the mean prevalence of shedders was around 9.5% for both scenarios 2A and 2B three years after the simulation started and increased to respectively 40.4% and 19.2% eight years after the simulation started. The mean number of yearly abortions increased from 0.4 and 0.54 the third year after the start of vaccination to 4.2 and 2.0 abortions per herd respectively during the eighth year after vaccination started.

### Influence of the ratio *p*_*v*_/*p *values on the model dynamics

As shown in Figure 2d and 2e for scenario 1, the mean prevalence of shedders was highly influenced by the values of the *p*_*v*_/*p *ratio (the ratio between the transition rate *SV*_*e *_=>*I*_*1*_*V*_*e *_and the transition rate *S *=>*I*_*1*_), whereas the mean yearly number of abortions (results not shown) and the mean dynamics of environmental bacterial load were not affected by this parameter. For *p*_*v *_= 0.9*p*, the mean prevalence of shedders was almost stable within the first two years of vaccination and decreased afterwards to reach 8.3% five years after the start of the simulation. On the contrary, when considering *p*_*v *_= 0.05*p*, the decrease was much faster and the mean prevalence of shedders was less than 1% five years after the start of the simulation. In both cases, the mean prevalence of shedders was less than 1% at the end of the simulation time.

### Influence of the vaccination scenarios and *p*_*v*_/*p *ratio on the extinction rate

**Figure 3:**
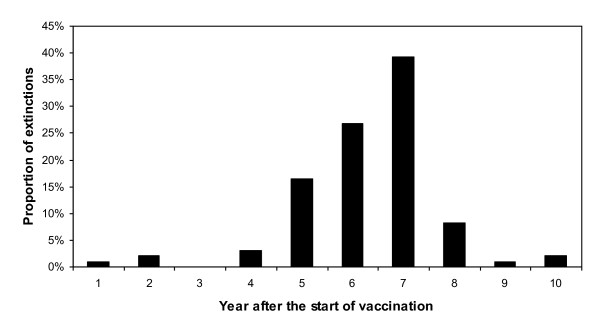
**Distribution of times to extinction of the 97 extinct trajectories of scenario 1**.

Whereas the extinction rate was nil when no control programme was implemented, it varied from 8% to 97% between the vaccination scenarios and the values of *p*_*v*_*/p *(Table 1). It appears that most of the extinctions occurred late: as shown in Figure 3 for scenario 1, 82.5% of the extinctions occurred between the 5^th ^and the 7^th ^year of the vaccination programme.

**Table 1:** Extinction rate and mean time to extinction for each of the vaccination scenarios

Criteria	Scenario						
	
	Control	1	1	1	2A	2B	3
		***p***_***v ***_**= 0.05*p***	***p***_***v ***_**= 0.21*p***	***p***_***v ***_**= 0.9*p***			
Extinction rate	0.00	1	0.97	0.84	0.08	0.43	0.95
Median time to extinction	-	week 272	week 312	week 390	week 138	week 276	week 348

Control: no control programme; scenario 1: vaccination of heifers and cows for a 10-year period; scenario 2: vaccination of heifers and cows for a 3-year period with (scenario 2A) or without (scenario 2B) loss of immunity one year after the last vaccination; scenario 3: vaccination of heifers for a 10-year period

## Discussion

In this study, we explored the long term effectiveness of three different vaccination strategies in a recently infected dairy cattle herd, through a modelling approach. Mathematical models are nowadays one of the most effective tools to compare control measures for both human [24-26] and animal infectious diseases [27-30]. Here, we focused on vaccination since it is widely used in the field and was identified as a long-term control option for *C. burnetii *infections by the EFSA [31]. Vaccination with a phase I vaccine in cattle was indeed shown to suppress shedding in milk, placenta, uterine fluid, vagina and colostrum [32,33]. In Rousset et al. [22], the vaccine appeared neither able to prevent infection in exposed kids, nor to clear infection in infected goats, but effectively reduced the level of shedding in a heavily infected herd. Hogerwerf et al. [34] also found that both the prevalence of shedders as well as the bacterial load in uterine fluid, vaginal swabs and milk were reduced in vaccinated dairy goats.

The vaccination scenarios tested in our study were (1) vaccination of the whole herd for 10 years, (2) vaccination of the whole herd for 3 years, and (3) vaccination of the heifers for only 10 years. Scenario 1 was predicted the most effective control strategy. In fact, all three vaccination strategies reduced the prevalence of shedders, the environmental bacterial load and the number of abortions. However, their effectivenesses were not equivalent. Since the infection was seldom eradicated in the first years of vaccination, an early cessation of vaccination (scenario 2) would be ineffective on the long run. Its short-term effect on infection dynamics depends on the duration of immunity for effectively vaccinated cows. According to Rodolakis et al. [35], in infected herds, more than 80% of the vaccinated cows still had immune markers one year after vaccination. However, at the same time, less than 60% of the vaccinated heifers were still skin-test positive. In the field, this means that immunity should last between one year (scenario 2A) and life long (scenario 2B). In this context, the increase in the prevalence of shedders, the environmental bacterial load and the number of abortions should not be observable in the first months following the cessation of vaccination. Nevertheless, the infection is spreading again. Thus, before stopping a vaccination programme on a farm, it seems essential to determine the presence or absence of *C. burnetii *in the herd. Diagnostic tests at a herd level (e.g. PCR in bulk tank milk) can probably be helpful [36], although they are imperfect.

According to our simulations, when only the heifers are vaccinated yearly (scenario 3), the decrease in the prevalence of shedders, the environmental bacterial load and the number of abortions is slower than when all the animals are vaccinated (scenario 1): it takes between 9 and 10 additional months to reach the same level of prevalence of shedders and environmental load, although the two strategies only differ in the initial action of the control programme. The extinction rate is high for both scenarios. Thus, although scenario 1 seems the best strategy from an epidemiological point of view, the difference between scenarios 1 and 3 is not so marked and a cost-benefit analysis would be useful to better compare the relative interest of these two strategies. It has to be highlighted that the numerical results of our study partially depend on the model structure and parameter values. The model represented the heterogeneity of shedding which is known to affect infection dynamics and hence the intervention efficacies in many diseases [37]. Indeed, sensitivity analysis show that model parameters governing the shedding levels, the characteristics of the bacterium in the environment as well as some of the physiological parameters strongly influenced the *C. burnetii *dynamics (see Additional File 1: section 2.1.). Here, parameter values were inferred or calibrated from field data of naturally infected dairy cattle herds [23]. Thus, we took into account the latest knowledge on *C. burnetii *infections. Although numerical values of the most influential parameters strongly influenced the output of numerical values, they did not change the relative ranking of the vaccination strategies. The only impact was that, for some combinations of parameter values, the differences between simulated effectiveness for the different vaccination scenarios became less marked, especially between scenarios 1 and 3.

The probability of infection for an effectively vaccinated susceptible cow *p*_*v *_was quantified based on the hazard ratio of the probability of shedding for vaccinated using non pregnant cows provided in Guatteo et al. [17]. Since the confidence interval of this parameter was wide, we studied the influence of this parameter value on the model outputs. Although the mean shedder prevalence was highly influenced by the *p*_*v*_/*p *ratio, the mean environmental bacterial load (which indirectly represents the infection risk for both animals and humans) decreased by roughly the same rate regardless of the ratio value. This is likely because the effectively vaccinated animals shed in decreased quantities. Therefore, irrespective of whether the mean prevalence of vaccinated shedders remains high, the prevalence of high shedders was reduced, with a major impact on the environmental load. This result has also been described by Lu et al. [38] who showed that to reduce the *Salmonella *prevalence in the long term, highly effective vaccines lowering the infectiousness would be a better choice than highly effective vaccines reducing susceptibility. Interestingly, the environmental bacterial load was hardly sensitive to the *p*_*v*_/*p *ratio (infection probability for effectively vaccinated cows), whereas the extinction rate was sensitive. Therefore, if the vaccine is to be used for eradication of *C. burnetii *from infected farms, both susceptibility and infectiousness of vaccinated animals have to be determined more accurately in order for the model to be used for prediction purposes or decision support. According to Rousset et al. [22], the lowest shedding level in vaginal swabs was shown to be more frequent in vaccinated than non vaccinated goats. However, further studies are needed to determine if a decrease of infectiousness is observed for all vaccinated animals or only for the animals vaccinated when non pregnant and still uninfected, and to quantify this decrease in all the shedding routes.

It should be noted that the extinction rate is highly influenced by the effect of vaccination on the susceptibility, the level of shedding and the mortality rate of the bacterium in the environment (see Additional file 1: section 2.1.2.), which are all not accurately documented variables. This extinction rate should then be interpreted with caution and used to compare different control strategies within the model. However, the behavior of the extinction rate suggests that it takes time to get free from *C. burnetii *within a herd.

This model was developed for dairy herds and the results presented here can not be straightforwardly generalized to small ruminants. A major difference is flock management. The typical size of a dairy flock is often much higher than the typical size of a dairy herd and kidding is usually synchronized. Therefore, the way to represent demography in the model should be adapted when representing *C. burnetii *spread in small ruminants. Besides, shedding characteristics and clinical manifestations may be different between species: according to Rodolakis et al. [15], ewes were found to shed mostly in faeces and vaginal mucus while goats seem to shed mostly in milk. Arricau-Bouvery et al. [21] reported that high abortion rates were rare, except in some caprine herds. However, all considered, our model represents an extensively documented basis for further development.

In conclusion, although an additional cost-benefit analysis considering the economic aspects of control programmes is needed to design an optimal control strategy, our modelling approach showed that a long term yearly vaccination would reduce infection risk in vaccinated herds.

## Competing interests

The authors declare that they have no competing interests.

## Authors' contributions

AC participated in the design of the study, carried out the model development and analysis, and drafted the manuscript. LH, DK and MN participated in the design of the study and helped to draft the manuscript. FB and EV participated in the design and coordination of the study, and helped to draft the manuscript. All authors read and approved the final manuscript.

## Supplementary Material

Additional file 1 1. Additional information on the model; 2. Sensitivity analysis Click here for file
