# Supplementary material for: Modelling effectiveness of herd level vaccination against Q fever in dairy cattle
Source: Vet Res. 2011 May 23;42(1):68. doi: 10.1186/1297-9716-42-68 (PMC3125226; doi:10.1186/1297-9716-42-68)
Supplement: Additional file 1 — 1. Additional information on the model; 2. Sensitivity analysis [file 1297-9716-42-68-S1.DOC]

Additional file

**1. Additional information on the model**

We modelled the spread of *C. burnetii* within a dairy cattle herd in order to compare the effectiveness of different vaccination strategies. Here the epidemiological and demographical parameters of the model are detailed, and the computation of the environmental bacterial load along time.

- 1. **Definitions and values of all the parameters of the model**

Table S1: Definitions of the epidemiological model parameters and their values used for simulations.

| **Parameter** | | **Definition** | **Value** | **Source** |
| --- | --- | --- | --- | --- |
| *m* (week^-1^) | | Transition rate *I_1_* => *S* and *I_1_V_e_* => *SV_e_* | 0.7 | Courcoul et al.  [1] |
| *q* (week^-1^) | | Transition rate *I_1_* => (*I_2_* or *I_3_*) and *I_1_V_e_* => (*I_2_V_e_* or *I_3_V_e_*) | 0.02 |
| *plp* | | Proportion of cows going from *I_1_* to (*I_2_* or *I_3_*) and becoming *I_3_* and going from *I_1_V_e_* to (*I_2_V_e_* or *I_3_V_e_*) and becoming *I_3_V_e_* | 0.5 | Calibrated to match the average number of *I3*cows observed in the field (R. Guatteo 2009, personal communication) |
| *r_1_* (week^-1^) | | Transition rate *I_2_* => *C_1_* and *I_2_V_e_* => *C_1_V_e_* | 0.2 | Courcoul et al.  [1] |
| *r_2_* (week^-1^) | | Transition rate *I_3_* => *C_1_* and *I_3_V_e_* => *C_1_V_e_* | 0.02 | Assumption that *I3* shed 10 times longer than *I2* |
| *s* (week^-1^) | | Transition rate *C_1_* => *I_2_* and *C_1_V_e_* => *I_2_V_e_* | 0.15 | Courcoul et al.  [1] |
| *τ*  (week^-1^) | | Transition rate *C_1_* => *C_2_* and *C_1_V_e_* => *C_2_V_e_* | 0.0096 | Based on Fournier et al. [2] and Plommet et al.[3], assumption that the mean life duration of antibodies in cattle is 2 years |
| *μ (*week^-1^) | | Mortality rate of *C. burnetii* | 0.2 | Courcoul et al.  [1] |
| *probav* | | Probability of abortion after a transition *S* => *I_1_*, *C_1_* => *I_2_* and *C_2_* => *I_2_* | 0.02 | Calibrated to match the distribution of abortions observed in the field (A.F. Taurel, 2010, personal communication) |
| *ρ^mf^* | | Proportion of bacteria shed through mucus/faeces filling the environmental compartment | 0.28 | Calibrated from expert opinion to match the environmental bacterial load inferred in Courcoul et al. [1] |
| ratio *ρ^milk^/ ρ^mf^* | | *ρ^milk^* = proportion of bacteria shed through milk filling the environmental compartment | 0.125 |
| *α* | milk | Probability distribution of the shedding routes for the *I_1_* cows | 0.31 | from field data (R. Guatteo 2009, personal communication) |
| mucus/feces | 0.62 |
| milk+ mucus/feces | 0.07 |
| *β* | milk | Probability distribution of the shedding routes for the *I_2_* cows after 4 weeks post-calving | 0.61 |
| mucus/feces | 0.33 |
| milk+ mucus/feces | 0.06 |
| *β_calv_* | milk | Probability distribution of the shedding routes for the *I_2_* cows in the 4 first weeks post-calving | 0.14 | from field data (R. Guatteo 2009, personal communication) |
| mucus/feces | 0.5 |
| milk+ mucus/feces | 0.36 |
| *γ* | milk | Probability distribution of the shedding routes for the *I_3_* cows after 4 weeks post-calving | 0.83 |
| milk+ mucus/feces | 0.17 |
| *γ_calv_* | milk | Probability distribution of the shedding routes for the I_3_ cows in the 4 first weeks post-calving | 0.25 |
| milk+ mucus/feces | 0.75 |
| *Q1* | low level | Probability distribution of the shedding levels for all the *I_1_* and for the *I_2_* shedding in mucus/faeces after 4 weeks post-calving | 0.85 |
| mid level | 0.15 |
| high level | 0 |
| *Q2* | low level | Probability distribution of the shedding levels for the *I_2_* shedding in milk after 4 weeks post-calving | 0.4 |
| mid level | 0.5 |
| high level | 0.1 |
| *Q3* | low level | Probability distribution of the shedding levels for all the *I_2_* in the 4 first weeks post-calving | 0.2 |
| mid level | 0.25 |
| high level | 0.5 |
| *Q4* | low level | Probability distribution of the shedding levels for the *I_3_* shedding in mucus/faeces after 4 weeks post-calving | 0.6 |
| mid level | 0.4 |
| high level | 0 |
| *Q5* | low level | Probability distribution of the shedding levels for all the *I_3_* shedding in milk and for the *I_3_* shedding in mucus/faeces in the 4 first weeks post-calving | 0.15 |
| mid level | 0.6 |
| high level | 0.25 |
|  |  |  |  |  |
| *Qty* (units of environment) | low level | Quantity of bacteria released by shedders in low, mid and high levels respectively | 1/3000 | Ratio between the 3 levels calculated from field data (R. Guatteo 2009, personal communication) |
| mid level | 1/30 |
| high level | 1 |
| *Q1V_e_* | low level | Probability distribution of the shedding levels for all the *I_1_V_e_* and for the *I_2_V_e_* shedding in mucus/faeces after 4 weeks post-calving | 1 | Based on Guatteo et al. [4] and Rousset et al. [5], assumption that the *Ve* animals shed less that the non *Ve* animals: no high level shedding and the probability to shed in mid level when *Q1* to *Q5* is now a probability to shed in low level |
| mid level | 0 |
| high level | 0 |
| *Q2V_e_* | low level | Probability distribution of the shedding levels for the *I_2_V_e_* shedding in milk after 4 weeks post-calving | 0.9 |
| mid level | 0.1 |
| high level | 0 |
| *Q3V_e_* | low level | Probability distribution of the shedding levels for the *I_2_V_e_* in the 4 first weeks post-calving | 0.5 |
| mid level | 0.5 |
| high level | 0 |
| *Q4V_e_* | low level | Probability distribution of the shedding levels for all the *I_3_V_e_* shedding in mucus/faeces after 4 weeks post-calving | 1 |
| mid level | 0 |
| high level | 0 |
| *Q5V_e_* | low level | Probability distribution of the shedding levels for all the *I_3_V_e_* shedding in milk and for the *I_3_V_e_* shedding in mucus/faeces in the 4 first weeks post-calving | 0.75 |
| mid level | 0.25 |
| high level | 0 |
| ratio  *p_v_/p* | standard value | Ratio between the transition rate *SV_e_* => *I_1_V_e_* and the transition rate *S*=>*I_1_* | 0.21 | Guatteo et al. [4] |
| bounds of the 95% CI tested for scenario 1 | 0.05 |
| 0.9 |

Table S2. Description of the model parameters for the herd demography and their values used for simulations.

| Parameters | | | Standard value |
| --- | --- | --- | --- |
| Replacement rate (year^-1^) | | | 0.355 |
|  | |  |  |
| Culling rate (week^-1^) | | lactation 1 | 0.0057 |
|  |  | lactation 2 | 0.0052 |
|  |  | lactation 3 | 0.0065 |
|  |  | lactation 4 | 0.0067 |
|  |  | lactations 5&6 | 0.0161 |
|  | |  |  |
| Probability distribution of the lactation numbers of the cows at the start of simulation | | lactation 1 | 0.337 |
|  |  | lactation 2 | 0.252 |
|  |  | lactation 3 | 0.173 |
|  |  | lactation 4 | 0.11 |
|  |  | lactation 5 | 0.088 |
|  |  | lactation 6 | 0.04 |
|  | |  |  |
| Calving-calving interval (weeks) | | | 55 |
|  |  | |  |
| Dry period (weeks) | | | 8 |
|  |  | |  |
| Non gestation period (weeks) | | | 15 |

**1.2. Computation of the environmental bacterial load**

For a given cow *i*, the quantity of bacteria arriving into the environment at time *t*, $Bact _{t}^{i}$, is:

$Bact _{t}^{i}=Qty_{i, t}^{milk}\rho^{milk}+Qty_{i, t}^{mf}\rho^{mf}$.

$Qty_{i, t}^{milk}$ is the quantity of bacteria shed in milk by the cow *i* at time *t*. It is equal to 1, 1/30, 1/3000 or 0 units of environment if the cow *i* is respectively a high level shedder in milk, mid level shedder in milk, low level shedder in milk or non shedder in milk at time *t*. $\rho^{milk}$ is the proportion of bacteria shed in milk arriving into the environment. $Qty_{i, t}^{mf}$ is the quantity of bacteria shed in mucus/faeces by the cow *i* at time *t*. It is equal to 1, 1/30, 1/3000 or 0 units of environment if the cow *i* is respectively a high level shedder in mucus/faeces, mid level shedder in mucus/faeces, low level shedder in mucus/faeces or non shedder in mucus/faeces at time *t*. If the cow *i* aborts at time *t*, an additional quantity of bacteria of 1 unit of environment (if the abortion occurs in the last third of gestation) or 1/30 unit of environment (if the abortion occurs in the first or second third of gestation) is shed in mucus/faeces by this cow. $\rho^{mf}$ is the proportion of bacteria shed in mucus/faeces arriving into the environment. This last parameter *ρ^mf^* is assumed to be higher than *ρ^milk^* (i.e. a lower proportion of the bacteria shed in milk is supposed to arrive into the environment of the herd, because most of the milk is directly sent to the bulk, and then to the dairy industry). At each time step and for each cow, $Qty_{i, t}^{milk}$ and $Qty_{i, t}^{mf}$ are randomly generated according to the probability distributions governing the shedding levels (*Q1* to *Q5* and *QVe_1_* to *QVe_5_*, different according to the type of shedder, the shedding route and the time after calving).

The total quantity of bacteria arriving into the environment at time *t* is then:

$Bact _{t}^{tot}=\rho^{milk}\sum_{i\in\left\{1...N_{t}\right\}} Qty_{i, t}^{milk}+\rho^{mf}\sum_{i\in\left\{1...N_{t}\right\}} Qty_{i, t}^{mf}$ with *N_t_* the total number of shedder cows in the herd at time *t*.

The global environmental bacterial load at time *t* is then: $E_{t+1}=E_{t}(1-\mu)+Bact_{t}^{tot}$.

**2. Sensitivity analysis**

In order to explore the impact of parameters and structural characteristics on the model outputs, a complete sensitivity analysis on the model variant without vaccination was performed. This study is presented in detail elsewhere [6]. Its most important results will be summarised here. Besides, to determine if variability in numerical values of the most influential parameters could impact the ranking of the studied vaccination strategies, an additional quick sensitivity analysis on the model with vaccination was also performed specifically in this study.

**2.1. Key points of the sensitivity analysis performed on the model without vaccination**

**2.1.1. Method**

The aim of the sensitivity analysis was to relate the variability obtained for the model outputs to that induced by the input parameters. The sensitivity of four outputs was evaluated for the 19 epidemiological parameters. The outputs, computed over a 5-year period, were the following: (i) the environmental bacterial load (ii) the prevalence of milk shedders, (iii) the prevalence of mucus/faeces shedders, and (iv) the number of abortions per herd per year. A fractional factorial experiment design of resolution V (allowing the exploration of the main effects and two-factor interactions) was used, with four parameter values per parameter related to the shedding and two parameter values for the other parameters. Four thousand ninety-six scenarios were run, each of them being characterized by a specific combination of parameter values. Since the model is stochastic, it was run 30 times for each combination of parameter values. A method developed by Lamboni et al. [7] was used and applied to the mean of the 30 repetitions of each scenario. This method allows simultaneously analyzing correlated variables (here the successive time points of a given output). It consists in two steps: a Principal Component Analysis (PCA) followed by an ANOVA. Sensitivity indices (SI), corresponding to the main effect or to interactions, and total sensitivities (TS), corresponding to the sum of the main effect and the interactions, were calculated for each factor.

**2.1.2. Results**

For the mean environmental bacterial load, the factors *Q1* (the probability distribution of the shedding levels for all the *I-* and for some *I+* shedding in mucus/faeces), *μ* (the mortality rate of *C. burnetii*) and *ρ^mf^* (the proportion of bacteria shed through mucus/faeces reaching the environment compartment) were the most influential ones. For the mean prevalences of mucus/faeces and milk shedders, the most sensitive factors were *q* (the transition probability from *I-* to *I+*), *s* (the transition probability from *C+* to *I+* representing the intermittency of shedding) and *Q1*, whereas the mean number of abortions was mostly impacted by *Q1*, *q* and less by *s*, *μ* and *ρ^mf^.*

**2.2. Key points of the sensitivity analysis performed on the model with vaccination**

**2.1.1. Method**

The impact of the five most influential factors identified in the sensitivity analysis of the model without vaccination, namely *Q1,* *q*, s, *μ* and *ρ^mf^*, on the ranking of the four studied vaccination strategies (1, 2A, 2B and 3) was specifically explored. A complete factorial experimental design was used with two levels per parameter (Table S3). Thirty repetitions for each of the 32 scenarios were run for every vaccination strategy. Three outputs were considered: (i) the environmental bacterial load, (ii) the prevalence of shedders, and (iii) the number of abortions per herd per year. The mean of each output over the 30 repetitions of a given scenario was computed over a 10-year period. For each scenario and each output, the four studied vaccination strategies were ranked (from 1 for the most effective strategy to 4 for the least effective strategy).

Table S3. Model parameters tested in the sensitivity analysis.

| Factor name | | Description | Standard value | Values tested  in the sensitivity analysis | |
| --- | --- | --- | --- | --- | --- |
|  |  |  |  |  |  |
| *q* (week^-1^) | | Transition rate *I_1_* => (*I_2_* or *I_3_*) and *I_1_V_e_* => (*I_2_V_e_* or *I_3_V_e_*) | 0.02 | 0.01 | 0.2 |
| *s* (week^-1^) | | Transition rate *C_1_* => *I_2_* and  *C_1_V_e_* => *I_2_V_e_* | 0.15 | 0.04 | 0.4 |
| *μ (*week^-1^) | | Mortality rate of *C. burnetii* | 0.2 | 0.08 | 0.5 |
| *ρ^mf^* | | Proportion of bacteria shed through mucus/faeces filling the environment compartment | 0.28 | 0.05 | 0.5 |
| *Q1* | low level | Probability distribution of the shedding levels for all the *I_1_* and for the *I_2_* shedding in mucus/faeces after 4 weeks post-calving | 0.85 | 0.85 | 0.15 |
|  | mid level |  | 0.15 | 0.15 | 0.6 |
|  | high level |  | 0 | 0 | 0.25 |

**2.1.1. Results**

Whatever the scenario and the output, the ranking of vaccination strategies was the same. Vaccination strategy 1 was the most effective, followed by strategies 3, 2B and at last 2A. However, the numerical values of the outputs and the differences between the four vaccination strategies were highly variable and a function of the combination of parameter values (Figure S1). As an example, in a non negligible number of cases (16 combinations of parameter values over 32), the decreases of the mean prevalences of shedders for scenarios 1 and 3 were very close.


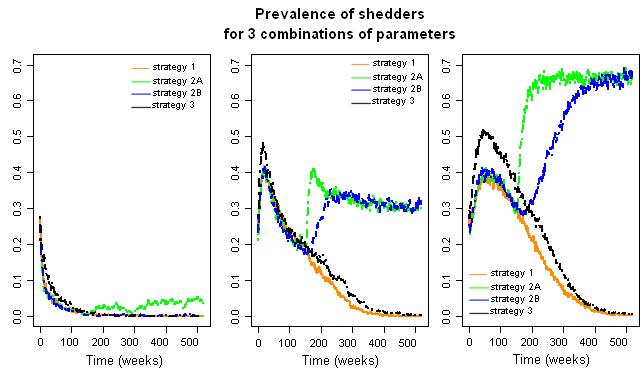


Figure S1. Temporal dynamics of the mean prevalence of shedders for the four vaccination strategies tested and for three distinct combinations of parameters *Q1,* *q*, s, *μ* and *ρ^mf^*. Combination 1: *q* = 0.01, *s* = 0.04, *μ* = 0.5, *ρ^mf^* = 0.05 and Q1 = (0.15,0.6,0.25); combination 2: same values except *q* = 0.2 and *μ *= 0.08 ; combination 3: *q* = 0.2, *s* = 0.4, *μ* = 0.5, *ρ^mf^* = 0.05 and Q1 = (0.15,0.6,0.25).

**REFERENCES**

[1] Courcoul A, Vergu E, Denis JB, Beaudeau F: **Spread of Q fever within dairy cattle herds: key parameters inferred using a Bayesian approach**. *Proc Biol Sci* 2010, **277**:2857-2865.

[2] Fournier PE, Marrie TJ, Raoult D: **Diagnosis of Q fever**. *J Clin Microbiol* 1998, **36**:1823-1834.

[3] Plommet M, Capponi M, Gestin J, Renoux G: **Fièvre Q expérimentale des bovins**. *Ann Rech Vet* 1973, **4**:325-346 (in French).

[4] Guatteo R, Seegers H, Joly A, Beaudeau F: **Prevention of Coxiella burnetii shedding in infected dairy herds using a phase I C. burnetii inactivated vaccine**. *Vaccine* 2008, **26**:4320-4328.

[5] Rousset E, Durand B, Champion JL, Prigent M, Dufour P, Forfait C, Marois M, Gasnier T, Duquesne V, Thiery R, Aubert MF: **Efficiency of a phase 1 vaccine for the reduction of vaginal Coxiella burnetii shedding in a clinically affected goat herd**. *Clin Microbiol Infect* 2009, **15**(Suppl 2):188-189.

[6] Courcoul A, Monod H, Nielen M, Klinkenberg D, Hogerwerf L, Beaudeau F, Vergu E: **Modelling of the heterogeneity of shedding in the within herd Coxiella burnetii spread and identification of related key parameters through a sensitivity analysis**. *J Theor Biol,* submitted.

[7] Lamboni M, Monod H, Makowski D: **Multivariate sensitivity analysis to measure global contribution of input factors in dynamic models**. *Reliab Eng & Syst Saf* 2011, **96**:450-459.
